# Apathy and impulsiveness in Parkinson disease: Two faces of the same coin?

**DOI:** 10.1097/MD.0000000000029766

**Published:** 2022-06-30

**Authors:** Rosanna Palmeri, Francesco Corallo, Lilla Bonanno, Simona Currò, Paola Merlino, Giuseppe Di Lorenzo, Placido Bramanti, Silvia Marino, Viviana Lo Buono

**Affiliations:** a IRCCS Neurological Center Bonino-Pulejo, Messina, Italy.

**Keywords:** anxiety, apathy, depression, dopamine, impulsiveness, Parkinson disease

## Abstract

Apathy and impulsiveness are 2 common non-motor symptoms in Parkinson disease that could occur in different periods or simultaneously. Apathy and impulsiveness could be interpreted as opposite extremes of a spectrum of motivated behavior dependent on dopaminergic dysfunction, in which, impulsivity, is a result of a hyperdopaminergic state, whereas apathy is viewed as a hypodopaminergic. The study aimed to investigate the presence of impulsiveness and other neuropsychiatric symptoms in Parkinson disease patients with apathy symptoms.

Eighty-one patients with Parkinson disease were enrolled in this retrospective study. All subjects were evaluated by the Italian version of the Dimensional Apathy Scale and the Barratt Impulsiveness Scale-version 11, to assess, respectively, apathy and impulsiveness; they were divided into 2 groups (apathy and no apathy). All patients were administered also with questionnaires assessing depressive and anxious symptoms.

Statistical analyses showed relevant results. In no-apathy group, education was a significant predictor on impulsiveness (attentional and motor) and apathy (executive and emotional); depression was a significant predictor on planning impulsivity and apathy.

This study aimed to consider the importance of apathy and impulsivity in Parkinson disease. Although these are considered as opposite extremes of a spectrum of motivated behavior dependent on dopaminergic dysfunction, these can also occur separately. Moreover, several variables could represent important predictors of apathy and impulsiveness, such as depression. Future investigations should deepen the role of other demographics and psychological variables.

## 1. Introduction

Apathy is a neurobehavioral syndrome that affects behavior, cognition, and emotion. It could be defined such as a decrease of motivation that results in a decrease of goal-directed behaviors.^[1]^ Conventionally, apathy is characterized by 3 subtypes: decrease in emotional resonance (reward deficiency syndrome), decrease in cognitive interests (executive dysfunction), and absence of cognitive initiation in mental processes (auto-activation deficit).^[2]^

In Parkinson disease (PD), apathy represents a common neuropsychiatric disturbance diagnosed in 20% to 36% of new-onset patients drug-naives.^[3]^ It is related to more severe motor impairment, worse executive functioning, and a higher risk of developing dementia than PD patients without apathy.^[4]^ Anatomically, the severity of apathy in PD is related to frontal gray matter and ventral striatal volume reductions.^[5]^ A cross-sectional study^[6]^ has identified that apathy is related to specific clinical and demographic correlates, individuating that men with PD and older had more probability to be apathetic.

Apathy is an isolated symptom in the 40% of PD: it could show without depression or cognitive symptoms (it is called “pure apathy”).^[7]^ The prevalence of “pure apathy” (i.e., apathy without comorbid depression and dementia) has been found to range from 3% to 47.9%^[8]^ Frequently, it is related to psychiatric symptoms, such as anxiety and depression: apathy and psychiatric symptoms are frequently comorbid in patients with PD.^[7]^

Another typical non-motor symptom is impulsivity. Impulsivity is “a failure to resist an impulse, drive or temptation to perform an act that is harmful to the person and others.”^[9]^ In PD, impulsiveness may relate to the disease itself or the effect of PD treatment. Apathy and impulsiveness could occur in an individual patient, simultaneously.^[10]^ These conditions could be interpreted as opposite extremes of a spectrum of motivated behavior dependent on dopaminergic dysfunction, in which, impulsivity, is a result of a hyperdopaminergic state within corticostriatal systems, whereas apathy is viewed as a hypodopaminergic state in this circuit.^[11]^

Only a few studies have focused on apathy and impulsiveness such as coexistent multifactorial constructs in neurodegenerative disorders^[12–15]^ and studies that may identify temporal relationships between apathy and possible predictors for apathy in PD are lacking.

Our retrospective study investigated whether patients with apathy experienced “alterations” in impulsivity compared to patients who were not apathetic. Then, we also tried to see if there is a relationship between apathy and neuropsychiatric symptoms.

## 2. Methods

### 2.1. Participants

We have enrolled a sample of 81 patients with idiopathic PD diagnosed as per the UK Parkinson’s Disease Society Brain Bank criteria.^[16]^ All subjects signed informed consent in accordance with the Declaration of Helsinki. The study was approved by Istituito di Ricovero e Cura a Carattere Scientifico (IRCCS) Centro Neurolesi “Bonino-Pulejo” Ethical Committee. The sample recorded a disease duration for about 10 years. According to the cutoff score of the Italian version of the Dimensional Apathy Scale (I-DAS) (see below), we have divided patients into 2 groups: 41 apathetic and 40 nonapathetic. All patients were treated with combinations of levodopa and dopaminergic agonists (L-dopa + DA). No one was taking any psychotropic medications that may cause apathy or impulsiveness. All of them were stable pharmacological treatments in the last 6 weeks. The severity and stage of PD were assessed by using the Unified Parkinson Disease Rating Scale (UPDRS)^[17]^ and Hoehn and Yahr scale (H&Y).^[18]^

Exclusion criteria were as follows:

- H&Y Stage ≥3.
- Mini-Mental State Examination (MMSE)^[19]^ to assess global cognitive functioning ≤26.
- presence of psychiatric or other neurological disorders.

These criteria are helpful to excluded other external causes such as dementia, that could compromises self-judgement. For the same reasons, inclusion criteria were as follows:

- Diagnosis of idiopathic PD.
- No psychiatric history or comorbidities.
- No pharmacological treatment for apathy.

### 2.2. Apathy and impulsiveness evaluation

All patients underwent the I-DAS^[20]^ and the Barratt Impulsiveness Scale-version 11 (BIS-11).^[21]^ I-DAS is a 24-item self-report questionnaire rated on a 4-point Likert scale which is divided into 3 subdomains: executive subscale, assessing apathetic impairments associated with planning, attention or organization; emotional subscale, assessing apathy linked to altered emotion integration and behavioral/cognitive initiation subscale assessing apathy associated with loss of self-generation of behaviors or cognition. A cutoff score ≥29 was used to identify apathetic PD patients.

BIS is a 30-item self-report questionnaire reflecting the multifactorial structure of impulsivity; outcome variables include attention, motor, self-control, cognitive complexity, perseverance, and cognitive instability subscores. Conventionally, a score between 70 and 75 individuate a pathological impulsivity trait, instead of a score >75 could show an impulse control disorder.

All patients completed The Beck Depression Inventory (BDI-II)^[22]^ and the Hamilton Rating Scale (HAM-A),^[23]^ questionnaires assessing, respectively, depressive and anxious symptoms. The minimum depression score for no-apathy group was 11, the maximum 25; in the apathy group, the minimum was 11, the maximum was 28. For anxiety, instead, the minimum score in no-apathy group was 8, the maximum score was 24; in apathy group, the minimum score was 11, the maximum was 23.

### 2.3. Statistical analysis

The Shapiro normality test was carried out to analyze the distribution of the variables. The groups were compared using an independent sample *t* test (unpaired) or the Wilcoxon signed-rank test. Correlations between clinical variables (BDI-II, HAM-A) and the subitems of the BIS and I-DAS for no-apathy group and apathy group were computed by Spearman coefficient. We performed a multiple regression analysis on subitem BIS and subitem of I-DAS (dependent variables). At first, we focused on the influence of demographic and clinical variables, by using patient’s age, education, BDI-II, and HAM-A scores as predictors. We applied a backward elimination stepwise procedure for the choice of the best predictive variables according to the Akaike information criterion (AIC). Analyses were performed using an open-source R3.0 software package (R Foundation for Statistical Computing, Vienna, Austria). A 95% confidence level was set with a 5% alpha error. Statistical significance was set at *P* < .05.

## 3. Results

**Figure 1.**
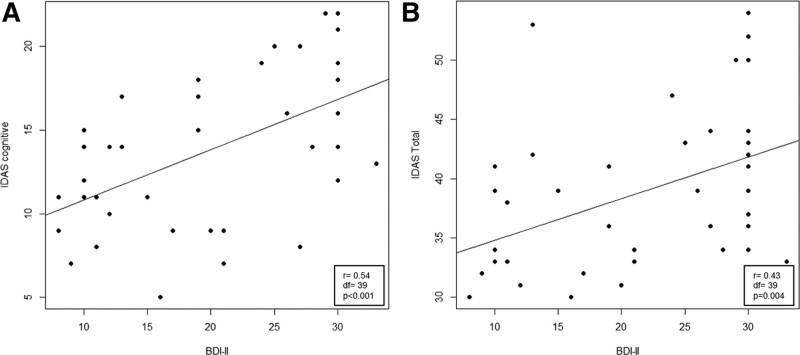
Correlation between BDI-II and I-DAS. (A) Scatter plot of I-DAS cognitive subscores and BDI-II. (B) Scatter plot of I-DAS total and BDI-II. BDI-II = Beck Depression Inventory, df = degrees of freedom, I-DAS = Italian version of the Dimensional Apathy Scale.

The 2 groups (41 subjects of apathetic and 40 subjects of not apathetic) did not differ on demographic and clinical aspects, MMSE scores, and mood evaluation (BDI-II and HAM-A). Demographic and clinical characteristics are shown in Table 1. Intergroup analysis (Table 1) showed significant difference in subitems of I-DAS, in particular, I-DAS executive (*P* < .001), I-DAS emotional (*P* < .001), I-DAS cognitive (*P* < .001), and I-DAS total (*P* < .001). A trend significative was found in BIS IDNP (*P* = .06). In no-apathy group, no significant correlation was found, while, in apathy group (Fig. 1) highlighted a positive correlation between BDI-II and I-DAS cognitive (*r* = 0.54; *P* < .001) and between BDI-II and I-DAS total (*r* = 0.43; *P* = .004). Moreover, no-apathy group highlighted a negative correlation between education and BIS IA (*r* = −0.35; *P* = .03), education and BIS IM (*r* = −0.43; *P* = .006), education and BIS total (*r* = −0.41; *P* = .01), and education and I-DAS executive (*r* = −0.32; *P* = .04). A significant positive correlation trend between education and I-DAS emotional (*r* = 0.30; *P* = .06) was found, while, in apathy group, no significant correlation was found. Multiple regression analyses showed significant predictors between the dependent variable and predictors. In no-apathy group (Table 2), education was a significant predictor on BIS IA (attentional), BIS IM (motor), and BIS total. Education is a predictor also on I-DAS executive, and I-DAS emotional score; BDI-II was a significant predictor on BID IDNP (nonplanning). Our patients have an average education of 10 years (Table 1), In the apathy group, BDI-II was a significant predictor of I-DAS cognitive and I-DAS total (Table 3).

**Table 1:** Demographic and clinical characteristics of 2 groups.

	No apathy (n = 41)	Apathy (n = 40)	
	Mean ± SD	Mean ± SD	*P* value
Age	69.21 ± 8.84	65.66 ± 8.80	.08*
Education	9.41 ± 4.41	10.07 ± 3.84	.33†
DD	8.23 ± 6.29	10.90 ± 9.23	.41†
MMSE	26.3 ± 2.93	25.66 ± 3.56	
BDI-II	18.08 ± 6.88	20.80 ± 8.39	.1†
HAM-A	16.92 ± 7.61	17.51 ± 6.78	.7†
BIS IA	16.31 ± 3.64	17.27 ± 3.12	.21*
BIS IM	22.26 ± 4.50	20.61 ± 4.25	.1*
BIS IDNP	26.08 ± 5.20	28.27 ± 5.25	.06*
TOT BIS11	64.38 ± 9.72	66.10 ± 10.40	.45*
I-DAS executive	8.23 ± 4.86	14.00 ± 4.47	<.001*‡
I-DAS emotional	7.15 ± 4.31	10.51 ± 3.91	<.001*‡
I-DAS initiation	6.79 ± 3.02	14.07 ± 4.65	<.001*‡
I-DAS tot	22.13 ± 5.18	38.59 ± 6.73	<.001*‡

BIS = Barratt Impulsivity Scale, DD = disease duration, MMSE = Mini-Mental State Examination, BDI = Beck Depression Inventory, HAM-A = Hamilton anxiety; BIS = Barratt Impulsivity Scale, IA = impulsivity attention, IDNP = impulsivity of (da in Italian) nonplanning, I-DAS = Italian dimensional Apathy scale, SD = standard deviation; SD = standard deviation.

* Unpaired Student *t* test.

† Mann–Whitney *U* test.

‡ *P* < .05.

**Table 2:** Backward linear regression significant predictors of BIS and I-DAS subscales in no-apathy group (n = 40).

Dependent variables	Predictors	β	Std β	*P* value	Adjusted *R*2
BIS IA	Education	−0.29	−0.35	.03	0.03
BIS IM	Education	−0.43	−0.42	.007	0.17
BIS IDNP	BDI-II	−0.25	−0.33	.06	0.1
BIS total	Education	−0.91	−0.41	.01	0.14
I-DAS executive	Education	−0.35	−0.32	.05	0.04
I-DAS emotional	Education	0.3	0.31	.06	0.03

β = regression coefficient, BIS = Barratt Impulsiveness Scale, BDI-II = Beck Depression Inventory, IA = impulsivity attention, IDNP = impulsivity of (da in Italian) nonplanning, I-DAS = Italian dimensional Apathy scale, Std β = standardized regression coefficient.

**Table 3:** Backward linear regression significant predictors of BIS and I-DAS subscales in apathy group (n = 41).

Dependent variables	Predictors	β	Std β	*P* value	Adjusted *R*2
I-DAS cognitive	BDI-II	0.34	0.61	<.001	0.24
I-DAS TOT	BDI-II	0.37	0.46	.01	0.14

β = regression coefficient; BIS = Barratt Impulsiveness Scale, BDI-II = Beck Depression Inventory, I-DAS = Italian dimensional Apathy scale, Std β = standardized regression coefficient.

## 4. Discussion

In this retrospective study, we evaluated apathy and impulsiveness in a sample of PD patients. These are 2 nonmotor symptoms common and often coexisting in PD.^[24]^ According to literature data,^[11–13]^ also our PD patients showed these symptoms, however, our data found no relationship between them into the 2 groups (apathy and no apathy). Indeed, in our sample, only a few patients were impulsive and they were equally distributed between apathy and no-apathy groups; moreover, “non-impulsive” patients were in great number in both groups; the apathy group seems to register a greater number of impulsive than no-apathy group, but the difference was not significant and had only qualitative value.

In the apathy group, there was a positive correlation between depression and total apathy, particularly for the cognitive (initiation) subdomain. Although our patients were not particularly depressed, higher levels of depression corresponded to higher levels of apathy. Depression was a risk factor for apathy. There is a great overlap between apathy and depression, with several common features: indeed, they are often misdiagnosed each other, which implies that these 2 mood disorders are also part of a spectrum of “hypodopaminergic.”^[25]^ In some PD patients, apathy may persist despite improvement in depressive symptoms due to the decreased functionality in the fronto-limbic brain circuits. Depression can include and develop into apathy, but apathy can also present itself as an isolated syndrome (pure apathy).^[26]^ Our data showed that depression could influence toplanning impulsiveness, corroborating the hypothesis that depression acts as a risk factor for the development of impulsiveness in PD patients. These results are biologically plausible, as depression and planning impulsiveness share several neurobiological mechanisms associated with reward.^[27]^

It is known that emotional deficits in PD patients were caused by the dysfunction of specific neurobiological circuits. Recently, Overton and Coizet^[28]^ proposed that anxiety could be attributable to a dysfunction of the superior colliculus one component of a rapid, reflexive threat detection system in the brain, consisting of the colliculus, pulvinar, and amygdala, which becomes hyper-responsive to sensory stimuli after dopamine denervation of striatum, typical in PD. Matsuura et al^[29]^ found that lower pulvinar intensity could determine a cognitive worsening, especially after surgical intervention of deep brain stimulation. Pulvinar seems to be involved also in contextual and multisensory processing and emotional response.^[30–32]^ Chou et al^[30]^ sustained pulvinar in involved in auditory processing, in particular, the study demonstrated that a multisensory bottom-up superior colliculus-pulvinar-auditory cortex pathway plays a role in contextual and cross-modality modulation of auditory cortical processing. Fang et al^[31]^ found the involvement of pulvinar and its projection to primary visual cortex in achieving context-dependent sharpening of visual representations. Similar data were found by Ibrahim et al.^[32]^ The pulvinar nucleus of the thalamus, indeed, is mutually connected with prefrontal cortex, sensory cortex, superior colliculus, and amygdala for its directly projections to these neurological structures.^[33]^ Zhou et al^[33]^ found the pulvinar is a hub linking the visual cortex with subcortical regions involved in the initiation and control of movement; indeed, pulvinar seems to be particularly important for coordinating body movements and visual perception. Our aim was to consider apathy and impulsiveness not as separate symptoms, but complementary to each other. However, our findings seem to not support the hypothesis that apathy and impulsiveness are 2 sides of the same coin. They may coexist, but they could show up more frequently separately. Probably the presence of one or the other is related to specific variables, anatomical and not. Indeed, anatomically, apathy and impulsiveness share the same neuronal circuits, the corticostriatal systems, and the same neurotransmitter: dopamine.^[11]^ Moreover, some demographic features could be related to some specific variables. Our findings demonstrated that education could interfere with impulsiveness, especially attentional and motor scores. Education is negatively related to executive and emotional apathy. A previous study found this relationship, without any difference in subgroups.^[34]^ Our data found that education is related to executive and emotional subcomponents of apathy.

Our subjects recorded more than 10 years of education; a previous study demonstrated that post-secondary education is negative related to increased BIS, but, in that case, education was related to attentional and nonplanning scores.^[35]^ Unfortunately, we have not found the influence exerted by other variables that could affect apathy and impulsiveness, such as, for example, relational and social aspects.^[6]^ Although our sample is numerically small, education is implicated and demonstrated the importance of non-neurological factors in the appearance of important nonmotor symptoms, such as apathy and impulsivity.

The small sample is the major limitation of this study; another limitation concerns the variables investigated. Moreover, it was very difficult to find PD patients with pure apathy. Further investigations should be aimed to select a group of PD patients with pure apathy excluding those with depression and/or anxiety symptoms, but also at finding risk factors, emphasizing the value of family, hobbies, personal interests, and sociocultural level.

This study aimed to consider the importance of apathy and impulsivity, 2 very common nonmotor symptoms in PD, which could both coexist but also be present separately. Both, however, should not be underestimated and above all, should not be confused with other symptoms of which they share some characteristics, as often happens with apathy and depression. The aim must be to produce an accurate diagnosis by identifying the right pharmacological intervention and not in order to improve the quality of life of patients with PD.

## Author contributions

Study design: Rosanna Palmeri, Francesco Corallo; study implementation: Rosanna Palmeri, Francesco Corallo, Viviana Lo Buono, Giuseppe Di Lorenzo, Silvia Marino; data collection and analysis: Rosanna Palmeri, Simona Currò, Paola Merlino, Lilla Bonanno; manuscript preparation: Rosanna Palmeri, Viviana Lo Buono, Francesco Corallo; approval of final version of this manuscript for publication: all authors.
